# Overexpression of a functional calcium-sensing receptor dramatically increases osteolytic potential of MDA-MB-231 cells in a mouse model of bone metastasis through epiregulin-mediated osteoprotegerin downregulation

**DOI:** 10.18632/oncotarget.16999

**Published:** 2017-04-10

**Authors:** Cédric Boudot, Lucie Hénaut, Ursula Thiem, Sandra Geraci, Mariangela Galante, Paulo Saldanha, Zuzana Saidak, Isabelle Six, Philippe Clézardin, Said Kamel, Romuald Mentaverri

**Affiliations:** ^1^ Inserm U1088, Centre Universitaire de Recherche en Santé, Université de Picardie Jules Verne, Amiens, France; ^2^ Inserm UMR 1033, LYOS, Lyon, France

**Keywords:** bone metastasis, breast cancer, calcium-sensing receptor, epiregulin, osteolysis

## Abstract

**Introduction and Aims:**

Osteolytic bone metastases are observed in advanced cases of breast cancer. *In vitro* data suggest that the activity of the calcium-sensing receptor (CaSR) expressed by metastatic cells could potentiate their osteolytic potential. This study aimed to demonstrate *in vivo* the involvement of the CaSR in breast cancer cells osteolytic potential and to identify potential targets linked to CaSR activity.

**Methods and Results:**

MDA-MB-231 stably transfected with plasmids containing either a full-length wild-type CaSR (CaSR-WT), or a functionally inactive dominant negative mutant (CaSR-DN) or an empty vector (EV) were intratibially injected into Balb/c-Nude mice. X-ray analysis performed 19 days after injection showed a dramatic increase of osteolytic lesions in mice injected with CaSR-WT-transfected cells as compared to mice injected with EV- or CaSR-DN-transfected cells. This was associated with decreased BV/TV ratio and increased tumor burden. Epiregulin, an EGF-like ligand, was identified by a DNA microarray as a possible candidate involved in CaSR-mediated osteolysis. Indeed, *in vitro*, CaSR overexpression increased both epiregulin expression and secretion as compared to EV- or CaSR-DN-transfected cells. Increased epiregulin expression was also detected in osteolytic bone lesions from mice injected with CaSR-WT-transfected MDA-MB-231. *In vitro*, exposure of osteoblastic cells (HOB and SaOS2) to exogenous epiregulin significantly decreased OPG mRNA expression. Exposure of osteoblastic cells to conditioned media prepared from CaSR-WT-transfected cells also decreased OPG expression. This effect was partially blocked after addition of an anti-epiregulin antibody.

**Conclusions:**

Overexpression of a functional CaSR in metastatic breast cancer cells dramatically amplifies their osteolytic potential through epiregulin-mediated OPG downregulation.

## INTRODUCTION

Breast cancer is the most common cancer among women worldwide. In these patients, metastatic cancer is the leading cause of death. Bones are a common place for breast cancer cells to metastasize. Within bone tissue, breast cancer cells activity favors osteolysis, which promotes hypercalcemia [1], bone pain and increases the risk of pathologic fractures. Breast cancer cells and bone cells communicate with each other through a vicious circle that results in progressive bone destruction and/or tumour growth [2]. Breast tumors from primary site produce parathyroid hormone related peptide (PTHrP) [1], which upregulates osteoblasts Receptor Activator of NFκB Ligand (RANKL) production. Binding of RANKL to its receptor RANK expressed on osteoclast precursor cells initiates their maturation and differentiation toward activated osteoclasts and stimlates bone resorption [3]. The calcium (Ca^2+^) released from bone resorption is thought to locally promote further PTHrP production from the metastatic tumour, thereby supporting the vicious circle [4]. Growth factors released from bone matrix destruction enable tumor cell survival and growth in bone microenvironment. This phenomenon amplifies the manifestation of bone metastases, which in turn increases the rate of bone turnover, thus feeding the vicious circle [2].

Initially cloned from parathyroid glands, where it is involved in systemic Ca^2+^ homeostasis [5], the calcium sensing receptor (CaSR) recently emerged as a new target within this vicious circle. Breast carcinomas express the CaSR. Of interest, CaSR expression is higher in breast cancer samples from patients with bone metastasis, suggesting that CaSR-positive tumors are more likely to metastasize to the skeleton [6]. In accordance with this hypothesis, expression of the CaSR is higher in breast cancer cell lines with relatively increased bone metastatic potential such as MDA-MB-231 [6]. Those cells also show a stronger CaSR-dependent migratory response to Ca^2+^ compared with cells with a lower metastatic potential (MCF-7, T47D) [7]. In malignant breast cells MCF-7 and MDA-MB-231, activation of the CaSR by Ca^2+^ stimulates PTHrP secretion [8]. Together, these data suggest that the large amounts of Ca^2+^ released after PTHrP-induced bone resportion could 1/ facilitate tumor cell migration into the bone through CaSR stimulation and 2/ participate to the vicious circle of bone resorption through CaSR induced-PTHrP secretion.

To date, our understanding on how the CaSR is involved in the development of osteolytic bone metastasis is limited to clinical observations and *in vitro* experimental data. Results obtained from animal models are lacking to truly demonstrate these concepts. This study aimed to i/ develop a model allowing an *in vivo* demonstration of the involvement of the CaSR on metastatic tumor osteolytic potential and to ii/ investigate if CaSR activity could influence osteolysis though a mechanism different from PTHrP modulation. For this purpose, we developed a model of MDA-MB-231 cells stably transfected to overexpress either a wild type or a dominant negative (R185Q) form of the CaSR. We demonstrated that the overexpression of a functional form of the CaSR favoured MDA-MB-231 osteolytic potential when intratibially injected to Balb/c-Nude mice. Studies lead by DNA microarray identified epiregulin, as a possible new candidate involved in CaSR-mediated osteolysis.

## RESULTS

### Stable transfection of human MDA-MB-231 breast cancer cells

**Figure 1:**
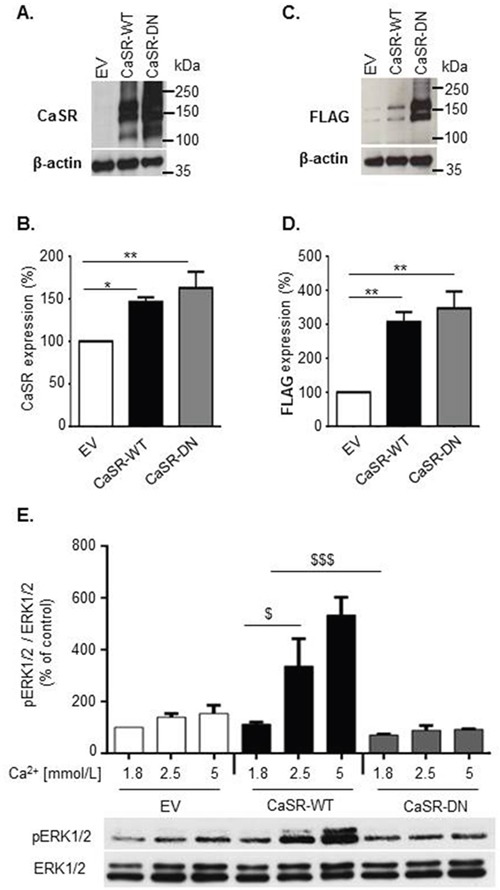
Expression and functionality of the CaSR in CaSR-WT-, CaSR-DN- and EV- transfected MDA-MB-231 **(A, B, C** and **D)** Evaluation of total (A and B) and FLAG-tagged (C and D) CaSR expression in EV-, CaSR-WT- and CaSR-DN-transfected MDA-MB-231 performed by western blot (A and C) and flow cytometry (B and D). **E.** Evaluation of ERK1/2 phosphorylation in MDA-MB-231 transfected with either EV, CaSR-WT or CaSR-DN and exposed for 10 min to increasing [Ca^2+^_0_] (1.8, 2.5 or 5 mM) (western blot). Results represent 3 independent experiments. *p<0.05,**p<0.01 vs. EV. $p<0.05, $$$p<0.001 vs. CaSR-WT cultured in 1.8 mM Ca^2+^.

To investigate the role of the CaSR on breast cancer cells osteolytic potential, we used MDA-MB-231 cells, an estrogen-independent breast cancer cell line able to colonize bone [9] and to induce osteolytic metastasis [10, 11]. MDA-MB-231 cells were stably transfected with plasmids containing either a full-length wild-type CaSR (CaSR-WT) or a functionally inactive dominant negative mutant (CaSR-DN). CaSR sequence was FLAG-tagged in its C-terminal region to discreminate between endogenous and genetically induced CaSR expression. Cells transfected with the empty pcDNA™3.1/Zeo^(+)^ plasmid (Empty Vector (EV)) served as controls. Western blot analyses performed with an anti-CaSR antibody demonstrated that both CaSR-WT- and CaSR-DN-transfected MDA-MB-231 displayed increased CaSR expression as compared with EV-transfected cells (Figure 1A). The same profile of expression was obtained on western blots performed with an anti-FLAG antibody (Figure 1C). Confirming these observations, a significant increase of both CaSR and FLAG expression was observed with flow cytometry in CaSR-WT and CaSR-DN-transfected MDA-MB-231 as compared with EV-transfected cells (Figure 1B and 1D).

Dominant-negative (DN) mutations disrupt the activity of wild-type genes [12]. CaSR activation results in ERK1/2 pathway activation in various cell types [13–15], including MDA-MB-231 cells [7]. Thus, we investigated CaSR functionality in EV-, CaSR-WT- and CaSR-DN-transfected cells through measurement of ERK1/2 phosphorylation levels after Ca^2+^ stimulation. Exposure of EV-transfected cells to increasing Ca^2+^ concentrations (from 1.8 to 5.0 mmol/L) promoted ERK1/2 phosphorylation in a concentration-dependent manner (Figure 1E). These effects were significantly amplified in CaSR-WT-transfected cells as compared with EV-transfected cells, while CaSR-DN-transfected MDA-MB-231 completely lost their abilities to respond to Ca^2+^.

### CaSR-WT overexpression enhances MDA-MB-231 osteolytic potential *in vivo*

**Figure 2:**
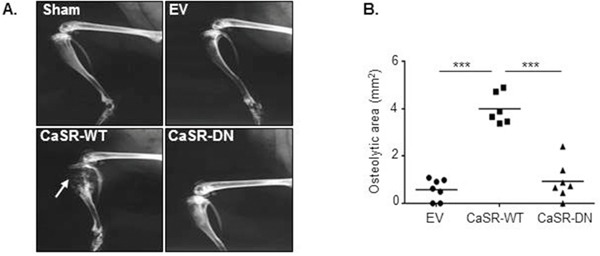
CaSR-WT overexpression favours MDA-MB-231 osteolytic potential *in vivo* **(A)** X-ray images of hind limbs taken 19 days after mice inoculation with EV-, CaSR-WT- or CaSR-DN-transfected MDA-MB-231. Arrow: osteolytic area. **(B)** Quantification of the osteolytic area. Sham was an age matched animal which had not been injected with tumor cells. ***p<0.001 vs. CaSR-WT.

**Figure 3:**
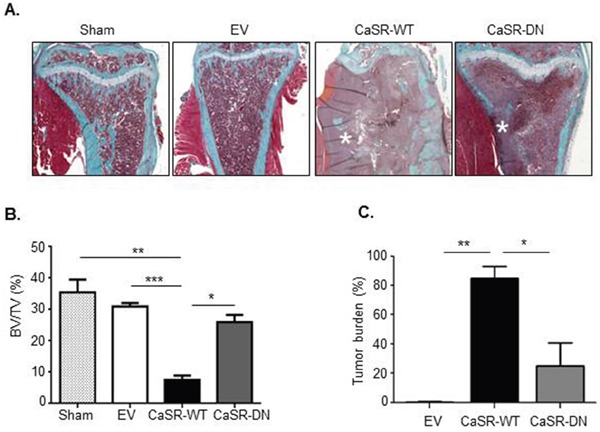
Intratibial injection of CaSR-WT-transfected MDA-MB-231 lowers BV/TV ratio **(A)** Micrographs of Goldner's trichrome-stained sections of tibial metaphysis. Goldner's trichrome staining was performed 19 days after intratibial injection. Green: bone – Red: bone marrow and tumor cells (asteriks). **(B)** Bone histomorphometry measurements (BV/TV, bone volume-tissue volume ratio). **(C)** Tumor burden. Sham was an age matched animal which had not been injected with tumor cells. *p<0.05, **p<0.01, ***p<0.001 vs. CaSR-WT-transfected cells.

To examine the influence of CaSR overexpression on MDA-MB-231 osteolytic potential, EV-, CaSR-WT- or CaSR-DN-transfected cells were intratibially injected into Balb/c-Nude mice. The development of bone lesions was followed by X-ray up to nineteen days. A sham operated mouse was used as a control. Data obtained by radiography demonstrated that 19 days after intratibial injection, the osteolytic area was around 4 fold wider in mice injected with CaSR-WT-transfected cells as compared with mice which were injected with EV-transfected cells (Figure 2A and 2B). The extent of osteolytic lesions was similar between mice injected with EV-transfected MDA-MB-231 and those injected with CaSR-DN-transfected cells. Histomorphometric analysis showed that mice intratibially injected with CaSR-WT- transfected MDA-MB-231 had significantly lower BV/TV as compared with mice intratibially injected with EV-transfected cells (p=0.0004) or CaSR-DN-transfected cells (p=0.02) (Figure 3A and 3B). There was no significant difference between BV/TV ratios of mice injected with CaSR-DN-transfected cells as compared with mice injected with EV-transfected cells (p=0.2). Skeletal tumor burden in the intratibial cavity of mice injected with CaSR-WT-transfected cells appeared significantly more pronounced as compared to mice injected with EV- or CaSR-DN-transfected cells, due to replacement of bone tissue and bone marrow with tumor cells (Figure 3C). No skeletal lesion was observed in the sham animals.

### CaSR overexpression is associated with increased epiregulin expression in MDA-MB-231 cells

**Figure 4:**
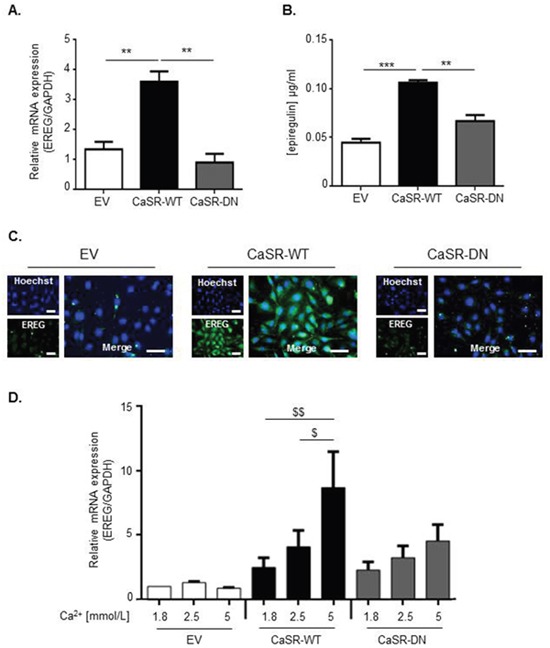
CaSR activity increases epiregulin expression and secretion by MDA-MB-231 cells *in vitro* **(A)** Evaluation of epiregulin (EREG) mRNA expression in either CasR-WT-, CaSR-DN- or EV-transfected cells (qRT-PCR). **(B)** ELISA performed on supernatants from EV-, CaSR-WT- and CaSR-DN-transfected MDA-MB-231 to detect epiregulin secretion. **p<0.01 or ***p<0.001 vs. CaSR-WT. **(C)** Immunocychemical evaluation of epiregulin protein expression in EV-, CaSR-DN or CaSR-WT MDA-MB-231. Scale bars: 50 μm. **(D)** Evaluation of epiregulin (EREG) mRNA expression in CasR-WT-, CaSR-DN- and EV-transfected cells exposed for 24 hours to 1.8, 2.5 or 5 mM of Ca^2+^. $p<0.05 or $$p<0.01 vs. cells exposed to 5 mM Ca^2+^ . Results represent 3 independent experiments.

We hypothesized that the high osteolytic potential of CaSR-WT-transfected MDA-MB-231 could be the consequence of an altered expression pattern of bone-regulatory genes, other than PTHrP. Thus, a transcriptomic analysis was performed using RNA microarray in order to compare CaSR-WT- and EV-transfected cells. Out of more than 44000 elements assessed, only 34 genes were differentially expressed in response to CaSR overexpression: 24 genes were up-regulated (Supplementary Table 1) and 10 genes were down-regulated (Supplementary Table 2). Among them, epiregulin (EREG), a known EGF-like ligand, showed a 3 fold up-regulation. Data obtained by quantitative RT-PCR and immunocytochemistry confirmed that epiregulin expression was significantly increased in CaSR-WT-transfected cells as compared with EV- or CaSR-DN-transfected cells (Figure 4A and 4C). No difference was observed between epiregulin transcripts levels from EV- and CaSR-DN-transfected cells. ELISA performed on supernatants showed increased epiregulin secretion in CaSR-WT-transfected cells as compared with EV- or CaSR-DN-transfected cells (Figure 4B).

To confirm the link between CaSR activity and epiregulin production, CaSR-WT-, CaSR-DN- and EV-transfected cells were exposed for 24 hours to increasing Ca^2+^ concentrations (1.8, 2.5 and 5 mmol/L) *in vitro* and epiregulin gene expression was assessed by qRT-PCR. In our model, Ca^2+^ significantly increased epiregulin gene expression in CaSR-WT-transfected MDA-MB-231 in a dose dependent manner, with a 4-fold increase observed in cells exposed to 5 mmol/L of Ca^2+^ (Figure 4D). Epiregulin expression was not significantly modulated in response to Ca^2+^ neither in EV-, nor in CaSR-DN-transfected MDA-MB-231.

**Figure 5:**
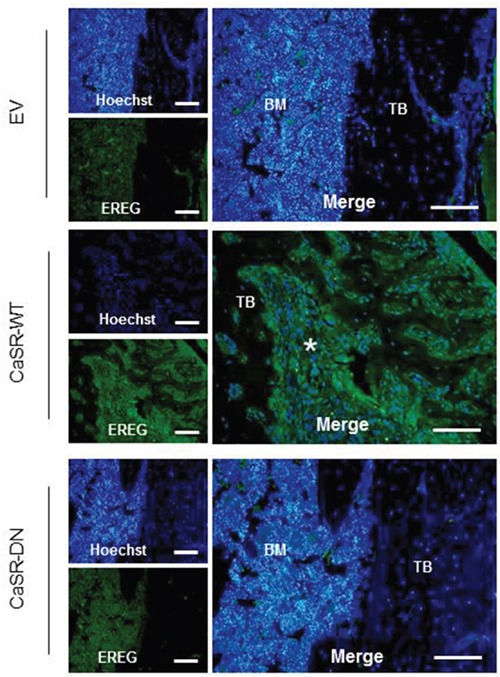
Epiregulin expression is increased in osteolytic bone lesions from mice injected with CaSR-WT-transfected MDA-MB-231 Immunohistochemical evaluation of epiregulin protein expression in bone sections from mice injected with EV-, CaSR-DN- or CaSR-WT-MDA-MB-231. BM: bone marrow, TB: trabecular bone, Asterisk: positive staining for epiregulin within invaded osteolytic bone lesions. Scale bars: 100 μm.

Data obtained by immunohistochemistry demonstrated an increased epiregulin expression within osteolytic bone lesions from mice injected with CaSR-WT-transfected MDA-MB-231 compared with bones from mice injected with either EV- or CaSR-DN-transfected cells (Figure 5).

### Epiregulin decreases OPG but not RANKL expression in human osteoblastic cells

**Figure 6:**
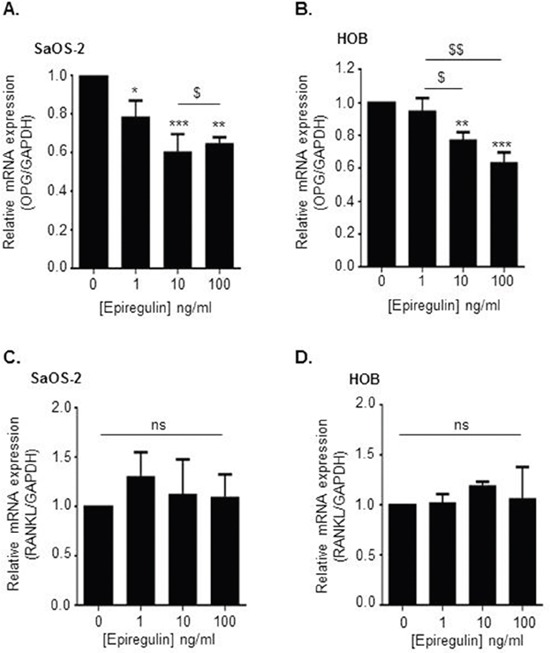
Epiregulin decreases OPG but not RANKL expression in osteoblastic cells SaOS2 and HOB **(A, B, C** and **D)** Evaluation of osteoprotegerin (OPG) (A and B) and RANKL (C and D) mRNA expression in SaOS2 (A and C) and HOB (B and D) cells exposed for 24 hours to recombinant epiregulin (qRT-PCR). **p<0.01 or ***p<0.001 vs control condition (without epiregulin). $p<0.05 or $$p<0.01 vs. cells exposed to 1ng/mL of epiregulin. Results represent 3 independent experiments.

EGF-like ligands were recently reported to promote osteoclastogenesis by decreasing OPG expression in osteoblastic cells, without modulation of RANKL expression [16]. We thus studied the effects of epiregulin on the RANKL/OPG ratio in two types of osteoblastic cells: the human osteoblastic cell line SaOS2 and the primary human osteoblastic cells HOB. Data obtained by qRT-PCR demonstrated that exposure of both SaOS2 and HOB cells to increasing concentrations of exogenous epiregulin decreased OPG gene expression in a concentration dependent manner (Figure 6A and 6B), without modulation of RANKL expression (Figure 6C and 6D).

**Figure 7:**
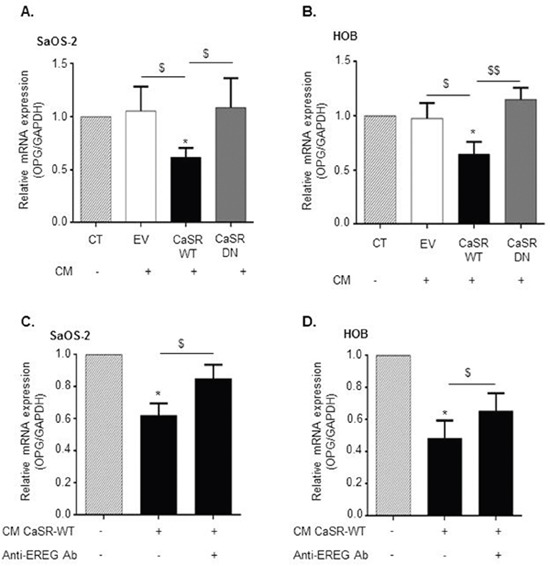
CaSR-mediated epiregulin secretion by MDA-MB-231 decreases OPG expression in osteoblastic cells **(A** and **B)** Evaluation of OPG mRNA expression in SaOS2 (A) and HOB (B) cells exposed for 24 hours to conditioned media (CM) prepared after exposure of CaSR-WT-, CaSR-DN- or EV-transfected MDA-MB-231 to 5 mM of Ca^2+^_0_ (qRT-PCR). *p<0.05 vs control condition (CT : 5 mM of Ca^2+^_0_). $p<0.05 or $$p<0.01 vs. CM from CaSR-WT-transfected cells. **(C** and **D)** Evaluation of OPG mRNA expression in SaOS2 (C) and HOB (D) cells exposed for 24 hours to CM from CaSR-WT-transfected MDA-MB-231, with or without anti-epiregulin antibody (qRT-PCR). *p<0.05 vs untreated cells. $p<0.05 vs. CM from CaSR-WT-transfected cells without anti-epiregulin antibody. Results represent 3 independent experiments performed in triplicate.

To ascertain that CaSR activation in tumor cells is responsible for decreased osteoblasts OPG production, SaOS2 and HOB cells were exposed for 24 hours to conditioned media prepared from CaSR-WT-, CaSR-DN- and EV-transfected cells exposed to 5 mmol/L of Ca^2+^ for 24 hours. Exposure of SaOS2 and HOB cultures to conditioned media prepared from CaSR-WT-transfected cells decreased OPG mRNA expression by approximately 50% as compared with EV- and CaSR-DN-transfected cells (Figure 7A and 7B). Exposure to conditioned media prepared from CaSR-DN and EV-transfected cells had no effect on HOB and SaOS2 OPG expression as compared with the control condition.

To evaluate if the decrease of OPG expression induced by conditioned medial from CaSR-WT-transfected cells depends on epiregulin secretion, the same procedure was reproduced using a neutralizing antibody targeting epiregulin. As demonstrated in Figure 7C and 7D, the use of the anti-epiregulin antibody partially blocked the decrease of OPG expression observed in response to a 24 hours exposure to conditioned media from CaSR-WT-transfected cells, in both SaOS2 and HOB cells.

## DISCUSSION

This report demonstrated that high expression of a functional CaSR conferred osteolytic advantage to MDA-MB-231 cells when intratibially injected to Balb/c-Nude mice *in vivo*. It also provided evidence that CaSR activation in MDA-MB-231 cells promoted the expression and secretion of epiregulin, an EGF-like ligand, which decreased OPG expression in osteoblastic cells.

Stable transfection of MDA-MB-231 cells with plasmids containing either a full-length functional wild-type CaSR (CaSR-WT) or a functionally inactive dominant negative mutant (CaSR-DN) or an empty vector, and their subsequent intratibial injection into Balb/c-Nude mice allowed us to create an accurate model to study the involvment of the CaSR in metastatic bone osteolysis. A dramatical osteolysis had been observed after injection with MDA-MB-231 transfected to overexpress a functional CaSR (CaSR-WT) as compared with mice injected with EV- and CaSR-DN-transfected cells. This suggests that a high level of functional CaSR may be necessary for metastatic breast cancer cells to promote bone osteolysis. These data support observations from Mihai *et al*., who reported that high tumor CaSR expression strongly associates with the presence of bone metastases [6]. Of note, skeletal tumor burden was more pronounced within the intratibial cavity of mice injected with CaSR-WT-transfected cells than in mice injected with EV- or CaSR-DN-transfected cells. This was associated with increased expression of Ki67, a cellular marker of proliferation, within osteolytic lesions from mice injected with CaSR-WT-transfected cells (Supplementary Figure 1). This suggests that the high amounts of Ca^2+^ released at sites of bone resorption might promote CaSR-induced MDA-MB-231 cell proliferation. However, data obtained *in vitro* showed that Ca^2+−^induced CaSR activation did not modulate neither the proliferation, nor the apoptosis of EV-, CaSR-DN- or CaSR-WT-transfected MDA-MB-231 cells (Supplementary Figure 2A and 2B.). To explain this phenomenon, we hypothesized that the osteolysis induced *in vivo* by the MDA-MB-231 overexpressing the functional CaSR might have favored the release of growth factors such as TGF-β from the bone matrix, which in turn might have favored tumor cell proliferation.

*In vitro*, activation of the CaSR with Ca^2+^, spermine, aminoglycoside antibiotics or allosteric (type II) calcimimetics increased PTHrP secretion by breast cancer cells [8, 17]. Since PTHrP secretion by metastatic cells increases osteoblasts RANKL production and subsequent osteclastogenesis, the current prevailing view is that CaSR osteolytic potential comes from its ability to increase metastatic breast cancer cells PTHrP secretion. We hypothesized that, apart from PTHrP secretion, high CaSR activity might modulate the expression pattern of other bone regulatory genes. Data obtained from comparative transcriptomic analysis identified epiregulin, which expression is tripled in CaSR-WT-transfected MDA-MB-31 as compared to EV-transfected cells, as a potential candidate.

Epiregulin belongs to the epidermal growth factor (EGF) family of peptide growth factors [18]. EGF-like ligands and their cognate receptors modulate cell functions in a variety of ways, including proliferation, survival, adhesion, migration and differentiation. In this work, data obtained *in vitro* confirmed that epiregulin expression and secretion were highly upregulated in CaSR-WT-transfected cells compared to CaSR-DN- or EV-transfected cells. This was associated with increased epiregulin detection in osteolytic lesions from mice intratibially injected with CaSR-WT-transfected MDA-MB-231 compared to mice injected with CaSR-DN- or EV- transfected cells. This phenomenon may result both from the high level of epiregulin expressed in CaSR-WT-transfected cells and the increased tumor burden observed in response to high CaSR activity. Confirming the link between CaSR activity and epiregulin gene expression, Ca^2+^ stimulation of the functional CaSR overexpressed in MDA-MB-231 cells concentration-dependently increased epiregulin mRNA expression while no difference was seen neither in EV-, nor in CaSR-DN-transfected MDA-MB-231.

In a recent study, Zhu *et al*. reported that EGF-like ligands strongly stimulate osteoclast formation in co-cultures of osteoblastic cells and bone marrow macrophages (BMMs) through the down-regulation of osteoblast OPG and MCP-1 [16]. In this study, co-cultures with MDA-MB-231 had similar effects on osteoblasts expression of OPG and MCP-1. Those effects could be partially abolished by EGFR inhibitor. Thus, the authors reasoned that EGF-like ligands, similarly to PTHrP, may increase osteolytic lesions [16]. In our model, overexpression of a functional CaSR amplifies MDA-MB-231 osteolytic potential and epiregulin secretion *in vivo*. We thus hypothesized that CaSR effects on MDA-MB-231 osteolytic potential could be the consequence of decreased osteoblasts OPG expression in response to increased epiregulin secretion by metastatic cells.

In line with this hypothesis, exposure of both HOB and SaOS2 cells to increasing doses of epiregulin decreased OPG gene expression in a concentration dependent manner. Confirming previous data obtained from Zhu et al. [16] we did not observe any modulation of osteoblasts RANKL expression in response to exogenous epiregulin. Conditioned media prepared from CaSR-WT-transfected cells decreased both SaOS2 and HOB OPG mRNA expression by approximately 50% as compared with EV- and CaSR-DN-transfected cells. The use of a neutralizing antibody targeting epiregulin partially blocked this phenomenon, demonstrating that CaSR-mediated decrease of OPG in osteoblastic cells partly depends on epiregulin secretion. Since CaSR activation on tumor cells promotes the secretion of PTHrP, which is also known to block OPG production [3], the fact that the neutralizing antibody targeting epiregulin does not totally block CaSR-mediated decrease in OPG expression is not surprising. In prostate cancer cells, CaSR stimulation transactivates the EGFR, leading to ERK phosphorylation and resultant PTHrP secretion [19]. Based on this observation, we cannot exclude in our study that CaSR-mediated epiregulin secretion might locally transactivate EGFR and improve PTHrP secretion by MDA-MB-231, which would indirectly worsen OPG inhibition in osteoblasts.

It would have been interesting to assess variations in osteoclasts number as a consequence of CaSR-induced osteolysis. However, in our model, the osteolytic activity of CaSR-WT-transfected MDA-MB-231 was so strong within 19 days, that the tumor invaded almost the whole bone. Therefore, there were almost no bone, and consequently no bone cells, left within those lesions. This would have biased the assessment of osteoclasts number.

**Figure 8:**
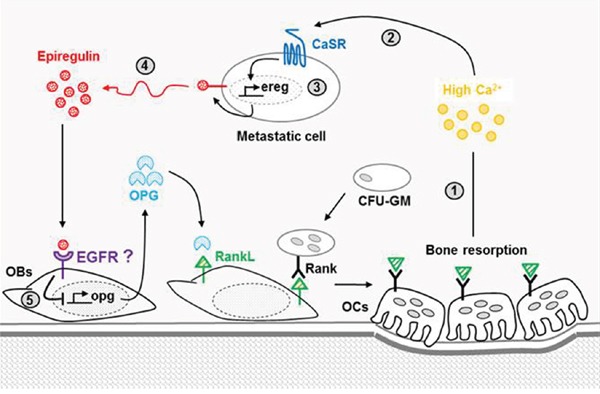
Involvement of the CaSR-epiregulin axis on the vicious circle of bone metastasis-induced osteolysis At sites of bone resorption, the high amounts of Ca^2+^_0_ released **(1)** can activate the CaSR expressed by breast cancer-derived metastatic cells **(2)**, which promotes epiregulin synthesis **(3)** and secretion **(4).** Epiregulin fixation on its receptors expressed by osteoblasts inhibits OPG synthesis **(5)**, thus favoring interactions between RankL expressed by osteoblasts and its receptor RANK expressed by immature osteoclasts. This favors osteoclastogenesis and in turn increases the rate of bone turnover, thus feeding the vicious circle.

To conclude, our data revealed an important role for the CaSR-epiregulin axis on metastatic breast cancer cells osteolytic potential. At site of bone resorption (Figure 8), the activation by Ca^2+^ of the CaSR expressed by metastatic breast cancer cells favors epiregulin synthesis and secretion, which in turn acts on osteoblastic cells to reduce OPG synthesis. By reducing the level of OPG released in bone microenvironment, the CaSR-epiregulin axis might increase bone resorption through increased osteoclastogenesis. EGFR inhibitors, such as cetuximab (which targets the receptor itself) or gefitinib and erlotinib (which target EGFR tyrosine kinase), were recently shown to efficiently reduce osteolytic lesions promoted *in vivo* by ADAMTS1 and MMP1 overexpressing MDA-MB-231, which secretes high amount of EGF like ligand. In line with this statement, significant relief of bone pain was observed in patients with bone metastasis in a clinical trial of the EGFR tyrosine kinase inhibitor. Together with these data, our work sheds lights on EGFR inhibitors as possible therapeutic strategies to prevent CaSR-mediated osteolytic bone metastasis.

## MATERIALS AND METHODS

### Cell lines and stable transfections

CaSR-WT and CaSR-DN cDNAs (CaSR R185Q) which have been described previously were used for the transfection of MDA-MB-231 cells (ATCC® HTB-26™) [20]. The coding regions of CaSR-WT and CaSR-DN containing a FLAG-tagged sequence were amplified by polymerase chain reaction (PCR) and subcloned at the HindIII/XbaI sites of the pcDNA3.1/Zeo(+) vector (V860-20, Invitrogen). MDA-MB-231 were transfected with pcDNA3.1/Zeo(+) vector containing CaSR-WT cDNA or CaSR-DN cDNA using Lipofectamine 2000 (catalog #11668-027, Invitrogen). MDA-MB-231 transfected with the empty vector (EV) were used as controls. Stably transfected cells were selected by growing them in zeocin-containing medium and cloned by limiting dilution. These cells were then routinely cultured in MEM (catalog #M4526, Sigma, St Louis, MO) supplemented with 10% fetal bovine serum (FBS), 1% GlutaMax, 1% of Penicillin-Streptomycin (complete MEM) and 0.8 mg/mL zeocin (catalog # R25005, Invitrogen). For the study of MAPK ERK activation, stably transfected MDA-MB-231 (150 000 cells/well; 6-well plates) were starved for 4 hours and stimulated for 10 minutes with increasing concentrations of Ca^2+^ (1.8, 3.0 or 5.0 mmol/L). Conditioned media (CM) mentioned in the manuscript were prepared by exposure of EV-, CaSR-WT- or CaSR-DN-transfected MDA-MB-231 to 5 mmol/L of Ca^2+^ for 24 hours.

The cell line of human osteosarcoma (SaOS2) was purchased from ATCC (ATCC® HTB-85™) and routinely cultured in complete DMEM (catalog #M5677, Sigma, St Louis, MO). Primary cultures of human osteoblasts (HOB) were purchased from Promocell (catalog #C-12720, Heidelberg, Germany) and cultured in Osteoblast Growth Medium (catalog # C-27001, Promocell, Heidelberg, Germany).

### Western blot analysis

Briefly, cells were washed once with PBS and homogenized at 4°C in a lysis buffer (10mM Tris-HCl, 150 mM NaCl, 10% glycerol, 2 mM Na3VO4 and protease inhibitors pH 7.4) containing either 1% NP40 (for the study of p42/44 proteins) or 1% SDS (for the study of CaSR expression). Proteins were then separated in 8 or 10% SDS-PAGE. After electrophoresis, samples were transferred to nitrocellulose membranes, blocked with 5% skimmed milk in TBS/0.1% v/v Tween 20 for 1 h, washed with TBS/Tween and incubated overnight at 4°C with primary antibodies : mouse monoclonal anti-CaSR (1:5000) (catalog #NB120-19347, Novus Biologicals), mouse monoclonal anti-FLAG M2 (1:2000) (catalog #200471, Agilent Technologies), rabbit polyclonal anti-phospho-ERK1/2 (1:1000) and rabbit polyclonal anti-ERK1/2 (1:1000) (catalog #9101 and #9102 respectively, Cell Signaling, Danvers, MA). Antibodies were diluted in 5% milk TBS/Tween. Blots were washed with TBS/Tween and incubated with appropriate horseradish peroxidase-conjugated secondary antibody (1:5000) (catalog #sc-2005 and #sc-2004 for anti-mouse and anti-rabbit respectively, Santa Cruz Biotechnology). After washing with TBS/Tween, blots were developed with the chemiluminescence method (ECL) (catalog #RPN2232, Amersham, Aylesbury, UK) and then probed with mouse monoclonal anti-β-actin antibody (1:2000) (catalog # A1978, Sigma, St Louis, MO) following the same method and levels of expression were corrected for minor differences in loading.

### Flow cytometry analysis

MDA-MB-231 were detached using cell dissociation solution (catalog # C5914, Sigma, St Louis, MO) and permeabilized using the Cytofix/CytopermTM Plus fixation/permeabilization kit (catalog #554715, BD Biosciences, Le Pont de Claix, France) following manufacturer's instructions. Cells were then incubated for 1 h at 4°C with primary antibody (mouse mAb anti-CaSR (5C10, ADD), catalog # ab19347, Abcam, 1:40 dilution from original unit); or mouse monoclonal anti-FLAG M2 (1:100) (catalog #200471, Agilent Technologies) or control isotype (catalog #X0943, Dako-Cytomation, Glostrup, Denmark), then washed in PBS containing 0.5% BSA, and labelled using polyclonal goat anti-mouse immunoglobulins/RPE (catalog # R0480, Dako-Cytomation, Glostrup, Denmark 1:50 dilution). Protein expression was assessed by flow cytometry using FACS CantoII (BD Biosciences, Le Pont de Claix, France). The results are presented in per cent of control condition (EV).

### Microarrays analysis

Total RNA from EV- and CaSR-WT-transfected MDA-MB-231 cells were isolated using RNeasy Mini Kit (catalog #74106, Qiagen France, Courtaboeuf). After treatment with turbo DNase (Life technologies, Illkirch, France), the quality of RNA samples was tested using RNA 6000 Nano kit and analyzed with Agilent 2100 Bioanalyser. Microarrays analysis was performed by PEGASE Biosciences (France) from Agilent Whole Human Genome 4×44K arrays.

### ELISA for epiregulin

Quantitative detection of soluble epiregulin was performed from supernatants of EV-, CaSR-WT- or CaSR-DN-transfected MDA-MB-231 using a commercially available kit (SEB945Hu 96 Tests, Cloud-Clone Corp.) according to manufacturer's instructions.

### Quantitative reverse transcription PCR (qRT-PCR)

Total RNA was extracted from EV-, CaSR-WT- or CaSR-DN-transfected MDA-MB-231 using the RNeasy Mini Kit (catalog #74106, Qiagen France, Courtaboeuf). RNA was reverse transcribed into cDNA using the “High Capacity cDNA Reverse Transcription Kit” (catalog #4368813, Applied Biosystems, Foster City, CA, USA) according to manufacturer's instructions. The qRT-PCR was performed on StepOnePlus real time PCR system (Applied Biosystems, Foster City, CA, USA) using gene-specific primers for epiregulin (catalog #QT00019194), OPG (catalog # QT00014294) or RANK-L (catalog # QT00215614) (QuantiTect Primer Assay, Qiagen France, Courtaboeuf). RNA expression of the different genes was corrected for GAPDH using the following primers: forward 5_atcaccatcttccaggagcga_3 and reverse 5_agccttctccatggtggtgaa_3.

### Animal studies

All procedures, including housing and care of the mice, euthanasia, and experimental protocols were conducted in accordance with a code of practice established by the local ethical committee of the University of Lyon (Lyon, France). This study was monitored on a routine basis by a veterinarian to ensure continued compliance with the proposed protocols.

Five-weeks-old female BALB/c homozygous (nu/nu) athymic mice were purchased from Charles River (Saint Germain sur l'Arbresle, France) and distributed in 3 groups (7 animals/group, plus a sham-operated mouse). After one week of acclimation, the mice were anesthetized by intraperitoneal injection of a solution (20 μl/g) containing 0,5 mg/mL of ketamine and 8 mg/mL of xylazin in saline solution (NaCl 0,9%) and received an intratibial injection of 100000 cells (EV, CaSR-WT and CaSR-DN) into the right hind limb. A Sham operated mouse was used as a control.

On days 7, 14 and 19 after tumor cell inoculation, radiographs of anesthetized mice were taken with the use of MIN-R2000 film (Kodak, Rochester NY) in an MX-20 cabinet X-ray system (Faxitron X-ray Corp, Wheeling, IL). Osteolytic lesions were identified on radiographs as radiolucent lesions in the bone. The area of the osteolytic lesions was measured using a Visiolab2000 computerized image analysis system (Explora Nova) and the extent of bone destruction per animal was expressed in square millimeters. Anesthetized mice were sacrificed after radiography on day 19 after tumor cell inoculation.

### Bone histomorphometry and histology

Besides radiography, tumor growth and osteolytic lesions were analyzed by histology. For this purpose, tibiae from animals were fixed, decalcified with 15% EDTA/ 0.4% PFA and embedded in paraffin. Section of 6-μm were stained with Goldner's Trichrome and used for histologic and histomorphometric analyses by means of a computerized image analysis system (Visiolab 2000). Histomorphometric measurements (i.e. bone volume to tissue volume [BV/TV] ratios) were performed in a standard zone of the tibial metaphysis, situated at 0.5 mm from the growth plate, including cortical and trabecular bones. The BV/TV ratio represents the percentage of bone tissue.

### Immunohistochemistry for epiregulin

Section of 6-μm were deparaffinized and incubated in sodium citrate (1M, pH=6) during 20 min at 100°C for antigen retrieval. Section were then fixed with 4% ice-cold paraformaldehyde (PFA) for 5 min at room temperature (RT), and quenched in 100 mmol/L glycin/PBS for additional 10 min. Tissues were then permeabilized for 10 min at RT with 0.1% triton X-100 in PBS containing 1% BSA. Non-specific binding of the antibody was blocked by incubation in a blocking solution (1% BSA in PBS) for 30 min at RT. Sections were then incubated overnight at 4°C with primary antibody (goat polyclonal IgG anti-epiregulin, R&D Systems, AF1195, 1:50 dilution from original unit) prepared in PBS containing 1% BSA. Bone sections were then rinsed and incubated with secondary antibody (AlexaFluor®488 Donkey anti-goat IgG Invitrogen A11055, 1:500 dilution from original unit), prepared in PBS 1% BSA, for 1 hr at RT. Samples were then widely washed in PBS. Nuclei were then counterstained with Hoechst and slides were mounted in Mowiol solution (Calbiochem®, Mowiol® 4-88) for fluorescent detection. The same protocol was performed to detect epiregulin expression on MDA-MB-231 monolayers on coverslips, except that the step of sodium citrate antigen retrieval was skipped.

### Statistical analysis

StatView v5.0 (version 5.0; SAS Institute, Inc.) was used for statistical analysis. Normally distributed data are presented as means ± Standard Error of the Mean (SEM) and non-normally distributed data as medians and minimum and maximum values. Kruskal Wallis test was used to compare the three groups for *in vivo* experiments. To account for multiple testing, Dunn's multiple comparison test was performed. All statistical tests were two-sided. For qRT-PCR and ELISA test results, a one-way ANOVA test was used to evaluate if differences between group means were statistically significant. P values of <0.05 were considered statistically significant.

## SUPPLEMENTARY FIGURES AND TABLES



## Abbreviations

CaSR: Calcium-sensing receptor
CaSR-WT: full-length wild-type CaSR
CaSR-DN: functionally inactive dominant negative mutant of the CaSR
EGF: epidermal growth factor
EV: empty vector
OPG: osteoprotegerin
PTHrP: parathyroid hormone related peptide
RANKL: receptor activator of NFκB ligand
RANK: receptor activator of NFκB ligand
