# Supplementary material for: Overexpression of a functional calcium-sensing receptor dramatically increases osteolytic potential of MDA-MB-231 cells in a mouse model of bone metastasis through epiregulin-mediated osteoprotegerin downregulation
Source: Oncotarget. 2017 Apr 10;8(34):56460–72. doi: 10.18632/oncotarget.16999 (PMC5593575; doi:10.18632/oncotarget.16999)
Supplement: Supplementary file 1 [file oncotarget-08-56460-s001.pdf]

Overexpression of a functional calcium-sensing receptor dramatically increases osteolytic potential of MDA-MB-231 cells in a mouse model of bone metastasis through epiregulin-mediated osteoprotegerin downregulation

SUPPLEMENTARY DATA

Assessment of cell survival (MTT)

MTT [3-(4,5-dimethylthiazolyl-2-yl)-2,5-diphenyltetrazolium bromide] (Sigma- Aldrich; St. Louis, MO) assay was used to evaluate the viability of EV-, CaSR-WT- and CaSR-DN-transfected MDA-MB-231 (8000 cells/well in 96 well plates) exposed for 24h to increasing Ca^{2+} concentrations (1.8, 2.5 or 5.0 $mmol.L^{-1}$ of Ca^{2+}_0). After stimulation, MTT solution was added to each well at a final concentration of 500 µg/ml for 2 h. Formazan crystals formed by living cells were then dissolved in DMSO and the absorbance was measured at 570 nm.

Assessment of early apoptosis and cell death

Annexin-V-FLUOS staining kit (Roche, #11858777001) was used to evaluate apoptosis. After adequate treatment, EV-, CaSR-WT- and CaSR-DN-transfected MDA-MB-231 (100 000 cells/well in 6 well plates) were trypsinized and stained with Annexin-V-FLUOS labeling solution according to manufacturer's instruction. Cells were analyzed using FACS Canto cytometer and FACS Diva Software (BD Biosciences).

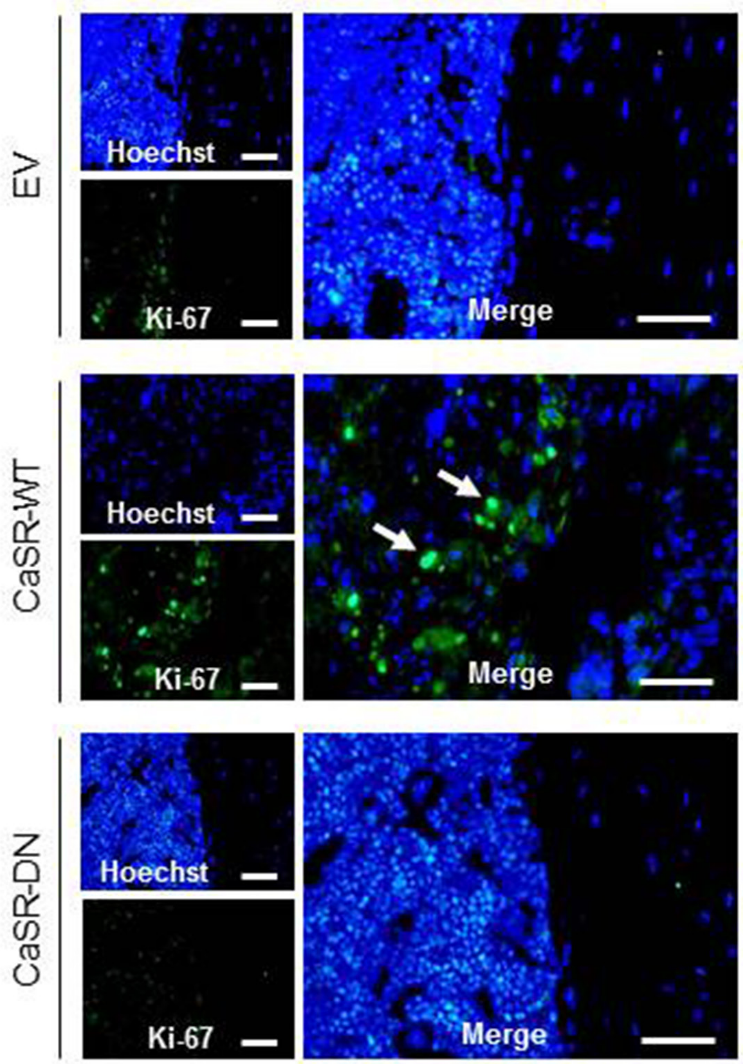

Supplementary Figure 1: Ki-67 expression is increased in osteolytic bone lesions induced by CaSR-WT-transfected MDA-MB-231. Arrows : Ki-67 positive staining. Scale bars : 50 µm.

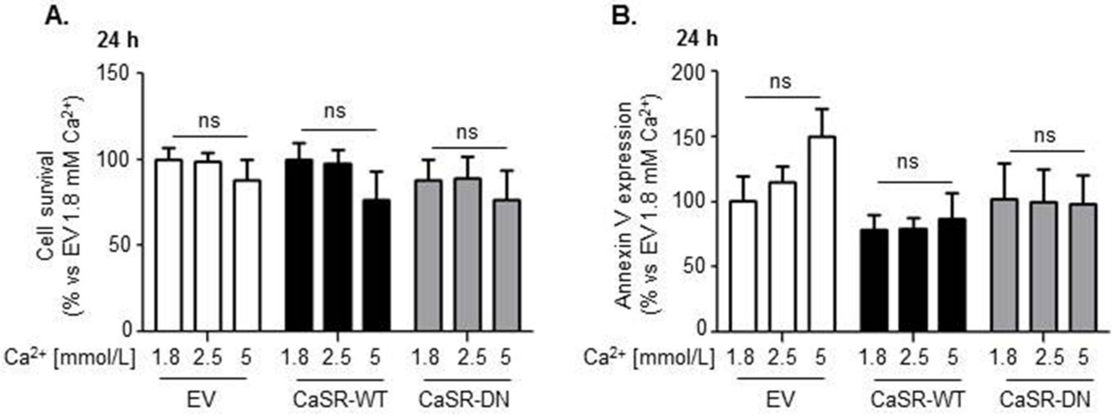

Supplementary Figure 2: CaSR activity does not modulate MDA-MB-231 survival, proliferation or apoptosis *in vitro*. **(A)** Data obtained from a MTT test performed on MDA-MB-231 cells exposed for 24 hours to increasing Ca^{2+} concentrations. **(B)** Data obtained from an Annexin V assay performed on MDA-MB-231 cells exposed for 24 hours to increasing Ca^{2+} concentrations. Results represent 3 independent experiments performed in triplicate.

Supplementary Table 1: Data obtained from a transcriptomic analysis performed on CaSR-WT- and EV-transfected MDA-MB-231

Symbol	Description	Fold change	*P value*
CFP	complement factor properdin	1.7602	0.0295
VEGFA	vascular endothelial growth factor A	1.7646	0.0229
HLA-E	major histocompatibility complex, class I, E	1.7869	0.0066
HLA-H	major histocompatibility complex, class I, H (pseudogene)	1.8128	0.0081
RAB18	RAB18, member RAS oncogene family	1.8255	0.0435
BMP1	bone morphogenetic protein 1	1.9008	0.0080
FLOT1	flotillin 1	1.9152	0.0060
HLA-C	major histocompatibility complex, class I, C	1.9303	0.0110
OAF	OAF homolog (Drosophilia)	1.9548	0.0167
FOS	FBJ murine osteosarcoma viral oncogene homolog	1.9628	0.0357
PAH	phenylalanine hydroxylase	1.9851	0.0060
HLA-B	major histocompatibility complex, class I, B	1.9874	0.0100
HLA-A	major histocompatibility complex, class I, A	2.0803	0.0121
ERRFI1	ERBB receptor feedback inhibitor 1	2.0839	0.0426
HOXA5	homeobox A5	2.1171	0.0070
TNNT2	troponin T type 2 (cardiac)	2.2314	0.0021
ZNF625	zinc finger protein 625	2.2796	0.0006
SIGIRR	single immunoglobulin and toll-interleukin 1 receptor (TIR) domain	2.3334	0.0060
C19orf60	chromosome 19 open reading frame 60	2.4808	0.0006
DDX26B	DEAD/H (Asp-Glu-Ala-Asp/His) box polypeptide 26B	2.5228	0.0010
UPP1	uridine phosphorylase 1	2.5355	0.0035
FGF13	fibroblast growth factor 13	2.5401	0.0121
TIMP1	TIMP metallopeptidase inhibitor 1	2.6858	0.0080
EREG	epiregulin	2.8395	0.0038
HOXA2	homeobox A2	3.4975	0.0006
VAV3	Vav 3 guanine nucleotide exchange factor	4.1518	0.0076

Table shows a list of the most upregulated genes in CaSR-WT- compared with EV-transfected MDA-MB-231.

Supplementary Table 2: Data obtained from a transcriptomic analysis performed on CaSR-WT- and EV-transfected MDA-MB-231

Symbol	Description	Fold change	*P value*
SLC25A25	solute carrier family 25	0.2542	0.0006
MED26	mediator complex subunit 26	0.3751	0.0035
KLHDC10	kelch domain containing 10	0.3869	0.0006
PCGF5	polycomb group ring finger 5	0.4709	0.0060
DPYSL3	dihydropyrimidinase-like 3	0.5324	0.0081
COL9A1	collagen, type IX, alpha 1	0.5460	0.0293
DYRK3	dual-specificity tyrosine-(Y)-phosphorylation regulated kinase 3	0.5520	0.0125
GRB14	growth factor receptor-bound protein 14	0.5565	0.0342
IFRD1	interferon-related developmental regulator 1	0.5612	0.0212
LRP4	low density lipoprotein receptor-related protein 4	0.5705	0.0100

Table shows a list of the most downregulated genes in CaSR-WT- compared with EV- transfected MDA-MB-231.
